# Linked color imaging improves visibility of reflux esophagitis

**DOI:** 10.1186/s12876-020-01511-9

**Published:** 2020-10-27

**Authors:** Tsutomu Takeda, Daisuke Asaoka, Daiki Abe, Maiko Suzuki, Yuta Nakagawa, Hitoshi Sasaki, Yoshihiro Inami, Muneo Ikemura, Hisanori Utsunomiya, Shotaro Oki, Nobuyuki Suzuki, Atsushi Ikeda, Noboru Yatagai, Hiroyuki Komori, Yoichi Akazawa, Kohei Matsumoto, Kumiko Ueda, Hiroya Ueyama, Yuji Shimada, Kenshi Matsumoto, Mariko Hojo, Taro Osada, Shuko Nojiri, Akihito Nagahara

**Affiliations:** 1 Department of Gastroenterology, Juntendo University School of Medicine, 2-1-1 Hongo, Bunkyo-ku, Tokyo, 113-8421 Japan; 2 Department of Gastroenterology, Juntendo Tokyo Koto Geriatric Medical Center, Tokyo, Japan; 3 Department of Medical Technology Innovation Center, Juntendo University School of Medicine, Tokyo, Japan

**Keywords:** Reflux esophagitis, Linked color imaging, Blue LASER imaging, Visibility, Inter-rater reliability, Color difference

## Abstract

**Background:**

With more prevalent gastroesophageal reflux disease comes increased cases of Barrett's esophagus and esophageal adenocarcinoma. Image-enhanced endoscopy using linked-color imaging (LCI) differentiates between mucosal colors. We compared LCI, white light imaging (WLI), and blue LASER imaging (BLI) in diagnosing reflux esophagitis (RE).

**Methods:**

Consecutive RE patients (modified Los Angeles [LA] classification system) who underwent esophagogastroduodenoscopy using WLI, LCI, and BLI between April 2017 and March 2019 were selected retrospectively. Ten endoscopists compared WLI with LCI or BLI using 142 images from 142 patients. Visibility changes were scored by endoscopists as follows: 5, improved; 4, somewhat improved; 3, equivalent; 2, somewhat decreased; and 1, decreased. For total scores, 40 points was considered improved visibility, 21–39 points was comparable to white light, and < 20 points equaled decreased visibility. Inter- and intra-rater reliabilities (Intra-class Correlation Coefficient [ICC]) were also evaluated. Images showing color differences (ΔE*) and L* a* b* color values in RE and adjacent esophageal mucosae were assessed using CIELAB, a color space system.

**Results:**

The mean age of patients was 67.1 years (range: 27–89; 63 males, 79 females). RE LA grades observed included 52 M, 52 A, 24 B, 11 C, and 3 D. Compared with WLI, all RE cases showed improved visibility: 28.2% (40/142), LA grade M: 19.2% (10/52), LA grade A: 34.6% (18/52), LA grade B: 37.5% (9/24), LA grade C: 27.3% (3/11), and LA grade D: 0% (0/3) in LCI, and for all RE cases: 0% in BLI. LCI was not associated with decreased visibility. The LCI inter-rater reliability was “moderate” for LA grade M and “substantial” for erosive RE. The LCI intra-rater reliability was “moderate–substantial” for trainees and experts. Color differences were WLI: 12.3, LCI: 22.7 in LA grade M; and WLI: 18.2, LCI: 31.9 in erosive RE (*P* < 0.001 for WLI vs. LCI).

**Conclusion:**

LCI versus WLI and BLI led to improved visibility for RE after subjective and objective evaluations. Visibility and the ICC for minimal change esophagitis were lower than for erosive RE for LCI. With LCI, RE images contrasting better with the surrounding esophageal mucosa were more clearly viewed.

## Background

The increased frequency of gastroesophageal reflux disease (GERD) has led to increasing incidences of Barrett's esophagus and esophageal adenocarcinoma [1]. It is thought that the frequency of Barrett's esophagus and esophageal adenocarcinoma cases will increase among young Japanese because of increasing cases of GERD, obesity, and lifestyle changes, and decreased infections by *Helicobacter pylori* [2]. Because the presence of GERD leads to a decrease in quality of life [3, 4], the accurate diagnosis of reflux esophagitis (RE) is important. Recently, the usefulness of image-enhanced endoscopy (IEE) in the diagnosis of RE has been described [5–8]. Although several reports described the precise diagnosis of RE using magnifying endoscopy [9–11], within the clinic making an easy and precise diagnosis is a high priority.

An innovation in the field of IEE, linked color imaging (LCI) can differentiate color variations in images of mucosal blood vessels by distinguishing various red regions. By overlapping narrow band images from both 410 nm and 450 nm with white light images produced using 450 nm, the brightness of images on the screen is retained although images are still enhanced. In this way, both discolored and red lesions are more easily identified, and the variations found in mucosal colors are distinguished.

LCI has recently been used in investigations involving gastric cancer [12–15], chronic gastritis [16, 17], colon cancer [18], Barrett's esophagus [19], and superficial esophageal squamous cell carcinoma [20, 21]. However, the visibility of RE using LCI and blue LASER imaging (BLI) has not been elucidated. Therefore, using endoscopy, we examined the visibility of RE using LCI and BLI compared with white light imaging (WLI).

## Methods

### Patients

In a retrospective clinical study undertaken in a single center, we investigated any improvement in the visibility of RE during endoscopy using LCI and BLI compared with WLI. Consecutive patients with RE were chosen after undergoing esophagogastroduodenoscopy (EGD) with WLI, LCI, and BLI, with the use of EG-L590WR, EG-L600WR7 or EG-L600ZW (Fujifilm Co., Tokyo, Japan) endoscopic systems, a video processor (AdvanciaHD VP-4450HD; Fujifilm Co.; Structure Emphasis: B6, Color Emphasis: C1) and light sources (LASEREO LL-4450; Fujifilm Co.), between April 2017 and March 2019 at Juntendo Tokyo Koto Geriatric Medical Center. Endoscopy was conducted on all patients while conscious and mostly in an outpatient setting. The gastroesophageal junction (GE–J) was imaged throughout the Inspiration of Air Phase. Circumferentially observed images were captured in proximity to the squamocolumnar junction (SC–J). Study participants were enrolled according to the following inclusion criteria: more than 20 years of age; and were consecutive patients with RE who received WLI, LCI, and BLI. Patients with the following criteria were excluded: had previously undergone esophageal surgery or gastrectomy, had used histamine 2-receptor antagonists or proton pump inhibitors in the past month, or if endoscopic examinations were difficult due serious heart, liver, or lung diseases. Additionally, patients were not included in the study if the GE–J was not observed when fully extended. Endoscopies were performed for a variety of reasons, including the presence of abdominal pain, for medical check-ups, the presence of anemia or GERD symptoms, and to follow up gastric ulcers. A JPEG format was chosen for images of acceptable quality. File sizes of about 100 Kb were prevalent for images, which had a 640 × 510-pixel array, and 24-bit color.

The ethics committee of Juntendo Tokyo Koto Geriatric Medical Center (No. 101-9) approved the protocol of this clinical study. Patients provided us with written, informed consent prior to undergoing EGD.

### Study protocol

Endoscopists, each experienced in at least 10,000 EGD procedures, conducted the endoscopies for this study. A Los Angeles (LA) classification system was used to categorize RE cases [22]. A modified LA classification system was used to categorize minimal change esophagitis (MCE) [23, 24]. Erosive reflux esophagitis (ERE) includes LA grades A to D. Three expert endoscopists (TT, DA, and HM) assessed and discussed WLI images until a consensus view was reached for each endoscopic finding. Lecture sets were used and consisted of 30 images from each endoscopic finding; these were shown to raters for assessment. Atrophic gastritis cases were grouped as open (O 1–3) or closed (C 1–3) types according to a Kimura–Takemoto classification system [25]. A hiatus hernia was defined as an apparent separation of the esophagogastric junction and diaphragm by > 2 cm. A positive result from a 13C-urea breath test and/or the detection of anti-*H. pylori* antibodies in serum meant a patient was positive for *H. pylori* infection. The 13C-urea breath test was used to define eradication in a patient as testing negative for *H. pylori* infection from 4 to 8 weeks after post-eradication therapy.

### Visibility scores

Ten endoscopists (five expert raters A–E: MH, DA, HS, TT, YN; and five trainee raters A–E: MS, AI, DA, MI, HU) compared WLI to LCI and BLI using 142 images from 142 patients. Each endoscopist viewed 10.3 × 12.9 cm images in a random order against a dark background in PowerPoint. WLI and LCI or BLI images were shown side by side. All images did not display clinical data or the date of capture. An assessment of visibility was repeated 2 months after the first assessment, although endoscopists were not informed that they were going to assess the same images a second time. Changes in visibility were assessed according to the following scoring system: 5, improved; 4, somewhat improved; 3, equivalent; 2, somewhat decreased; and 1, decreased. For total scores, 40 points was classified as improved visibility, a score of 21–39 points was comparable to white light, and < 20 points was assessed as decreased visibility. The Intra-class Correlation Coefficient (ICC) was used to indicate inter- and intra-rater reliability.

### Objective evaluation

L* a* b* (L* = light/dark; a* = red/green; b* = yellow/blue) color scores in a Commission Internationale de l'Éclairage (CIE) LAB color space system [26] after using Adobe® Photoshop CC 2019 were used to assess images as described previously [27]. A region of interest (ROI; 20 × 20 pixels) was demarcated in two places, respectively, for RE mucosa (ERE; reddish mucosal break area, MCE; whitish minimal change area) adjacent to the surrounding esophageal mucosa (concentrically right next to RE mucosa) and adjacent to the surrounding gastric mucosa (right next to the anal side of RE mucosa). Color values (*L*, *a*, *b*) in the ROI and the average were calculated from a histogram panel. *L, a,* and *b* represented color scores in Photoshop (Lab color unit). In CIELAB, *L, a,* and *b* color values were converted into L* a* b* color values using the formula: L* = *L* / 256 × 100, a* = a − 128, b* = b – 128 [28, 29]. For L* a* b* color spaces in a given ROI, color differences (ΔE* = [(ΔL*) ^2^ + (Δa*) ^2^ + (Δb*) ^2^]^1/2^) in pixel values were used to assess the visibility of each color image.

### Statistical analysis

A Wilcoxon rank sum test was used to assess statistically significant differences in visibility scores rated by trainees and experts, and ΔE* and L* a* b* color values between images. Statistical significance was considered at a *P* value of < 0.05.

Inter- and intra-rater reliability was tested using ICC with 95% confidence intervals (CIs). Where two or more coders were present, inter-rater reliability with regard to interval, ordinal, and ratio variables was commonly assessed using the ICC [30]. Reliability was classified as follows: “perfect” when ICC was 1.0, “excellent” when > 0.81, “substantial” when 0.80–0.61, “moderate” when 0.60–0.41, “fair” when 0.40–0.21, and “slight” when < 0.20 [31, 32]. For statistical analyses, SAS v. 9.4 (SAS Institute, Cary, NC, USA) was used.

The use of 10 assessors ensured sample sizes were adequate for estimations. A value of 0.7 was set for minimally acceptable reliability (ρ0) and 0.8 for expected reliability (ρ1). A difference at α = 0.05 and β = 0.2 was detected using a sample size of ~ 52 [33].

## Results

### Patient characteristics

Table 1 sets out the characteristics of study participants. With a mean age of 67.1 years (range: 27–89) for 142 participants, 63 were male and 79 were female. According to a modified LA classification system for RE, grade M: 52, grade A: 52, grade B: 24, grade C: 11 and grade D: 3 were observed. Hiatus hernia was found in 76.1% of participants. *H. pylori* infections were noted in 10 patients, while 91 were negative, and 41 were post-eradication. Seventy-six patients showed atrophic gastritis (closed type: 33, open type: 43) while 66 patients did not (Table 1).

**Table 1:** Baseline characteristics

Characteristics	Value
Sex (male:female)	63:79
Age in years, mean ± SD (range)	67.1 ± 11.8 (27–89)
Reflux esophagitis	Grade M: 52, Grade A: 52, Grade B: 24, Grade C: 11, Grade D: 3
Hiatus hernia	None: 34, present: 108
Atrophic gastritis	C-0: 66, C-1–3: 33, O-1–3: 43
*H. pylori*	Negative: 91, positive: 10, post-eradication: 41

*H. pylori Helicobacter pylori*, *SD* standard deviation

### Visibility scores

Comparisons of LCI or BLI and WLI with regard to the visibility scores of trainees, experts, and all endoscopists are shown in Table 2. The total visibility score for LCI for all endoscopists was 36.9, for trainee endoscopists it was18.2, and for expert endoscopists it was 18.7. The visibility scores between trainees and experts did not show a significant difference for both LCI and BLI. Improved visibility for LCI was achieved for grade M: 19.2% (10/52), grade A: 34.6% (18/52), grade B: 37.5% (9/24), grade C: 27.3% (3/11), grade D: 0% (0/3), and for erosive reflux esophagitis (ERE): 33.3% (30/90), for all endoscopists. Improved visibility with BLI was not achieved for all RE cases. Equivalent visibility using LCI was achieved for grade M: 80.8% (42/52), grade A: 65.4% (34/52), grade B: 62.5% (15/24), grade C: 72.7% (8/11), grade D: 100% (3/3), and ERE: 66.7% (60/90) for all endoscopists. Only the scores of some participants indicated reduced visibility (Table 3).

**Table 2:** Visibility scores of experts, trainees, and all endoscopists (mean ± SD)

Reflux esophagitis		Trainees (n: 5)						Experts (n: 5)						All (n: 10)	Trainees vs. Experts
		A	B	C	D	E	Total	A	B	C	D	E	Total	Total	*P* value
MCE	LCI	3.4	3.4	3.5	3.5	3.7	17.6 ± 1.8	3.1	3.8	3.2	3.7	4.3	18.1 ± 1.3	35.7 ± 2.6	n.s
	BLI	2.6	2.7	3.0	2.5	2.9	13.7 ± 1.3	2.9	3.0	2.2	2.8	2.9	13.9 ± 1.4	27.6 ± 2.1	n.s
ERE	LCI	3.7	3.8	3.7	3.4	4.1	18.6 ± 1.8	3.4	4.0	3.6	3.7	4.2	19.0 ± 1.9	37.6 ± 3.3	n.s
	BLI	2.7	2.7	2.6	2.8	2.9	13.7 ± 1.3	3.0	3.1	2.3	2.5	3.0	13.9 ± 1.3	27.6 ± 2.3	n.s
All	LCI	3.6	3.7	3.6	3.5	3.9	18.2 ± 1.9	3.3	3.9	3.5	3.7	4.2	18.7 ± 1.8	36.9 ± 3.2	n.s
	BLI	2.7	2.7	2.8	2.7	2.9	13.8 ± 1.4	3.0	3.0	2.3	2.6	3.0	13.8 ± 1.4	27.6 ± 2.2	n.s

*BLI* blue LASER imaging, *ERE* erosive reflux esophagitis (LA grades A–D), *LCI* linked color imaging, *MCE* minimal change esophagitis (LA grade M), *n.s.* not significant, *SD* standard deviation

**Table 3:** Evaluation of LCI and BLI for visibility

	Grade of reflux esophagitis						
	A	B	C	D	MCE	ERE	All
*Improved visibility n (%)*							
LCI	18 (34.6)	9 (37.5)	3 (27.3)	0	10 (19.2)	30 (33.3)	40 (28.2)
BLI	0	0	0	0	0	0	0
*Equivalent visibility n (%)*							
LCI	34 (65.4)	15 (62.5)	8 (72.7)	3 (100)	42 (80.8)	60 (66.7)	102 (71.8)
BLI	52 (100)	24 (100)	11 (100)	3 (100)	52 (100)	90 (100)	142 (100)
*Decreased visibility n (%)*							
LCI	0	0	0	0	0	0	0
BLI	0	0	0	0	0	0	0

*BLI* blue LASER imaging, *ERE* erosive reflux esophagitis (LA grades A–D), *ICC* intra-class correlation coefficient (95% confidence interval), *LCI* linked color imaging, *MCE* minimal change esophagitis (LA grade M)

Visibility scores for LCI were compared between LCI users (who had experienced using LCI over three months) and LCI non-users in a sub-analysis; five LCI users, raters: DA, MS, TT, YN, DA, and five LCI non-users, raters: MH, AI, HS, MI, HU were employed. A total visibility score of 19.0 was found for LCI users and 17.8 for LCI non-users; a significant difference (*P* < 0.001) was observed.

**Fig. 1:**
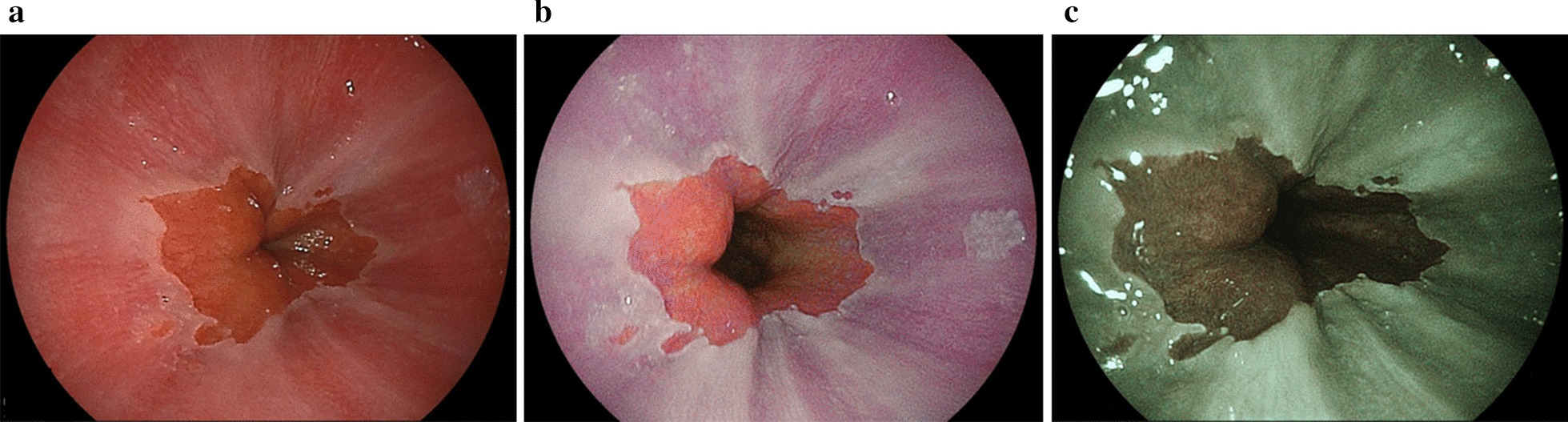
MCE using white light imaging, linked color imaging, and blue LASER imaging. **a** White light imaging (WLI). Minimal change esophagitis (MCE) with whitish turbidity. **b** Linked color imaging (LCI). The MCE was highlighted by a whitish color. The LCI image was scored as + 10 points representing improved visibility as evaluated by all endoscopists. **c** Blue LASER imaging (BLI)

**Fig. 2:**
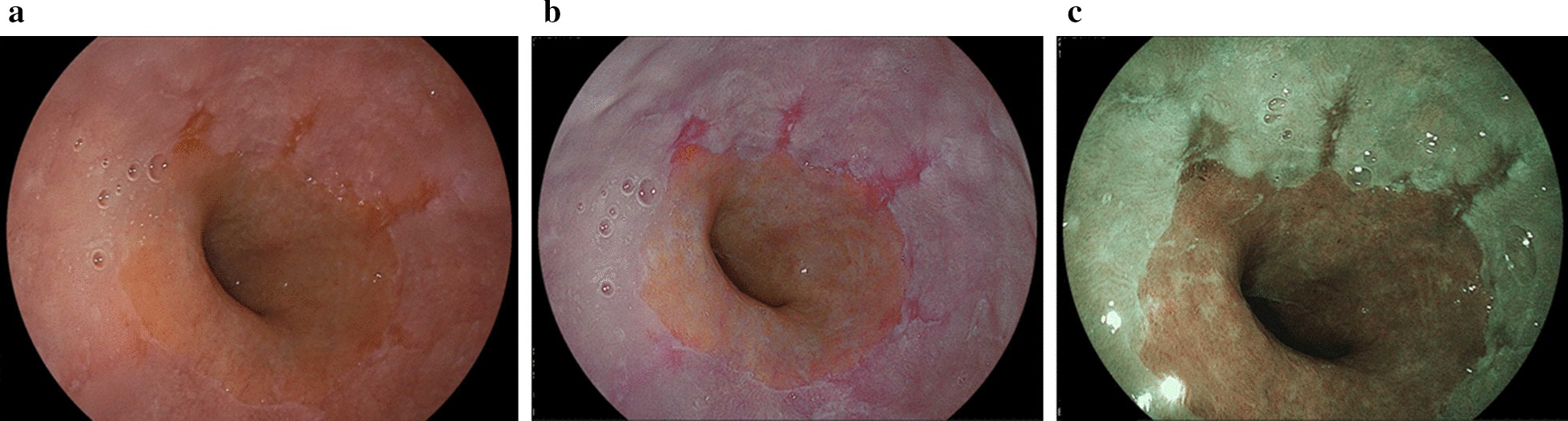
Reflux esophagitis (grade A) using white light imaging, linked color imaging, and blue LASER imaging. **a** White light imaging (WLI). Reflux esophagitis (LA grade A). **b** Linked color imaging (LCI). The reflux esophagitis was clearly detected and became highlighted in a red color. The LCI image was scored as + 15 points representing improved visibility as evaluated by all endoscopists. **c** Blue LASER imaging (BLI)

**Fig. 3:**
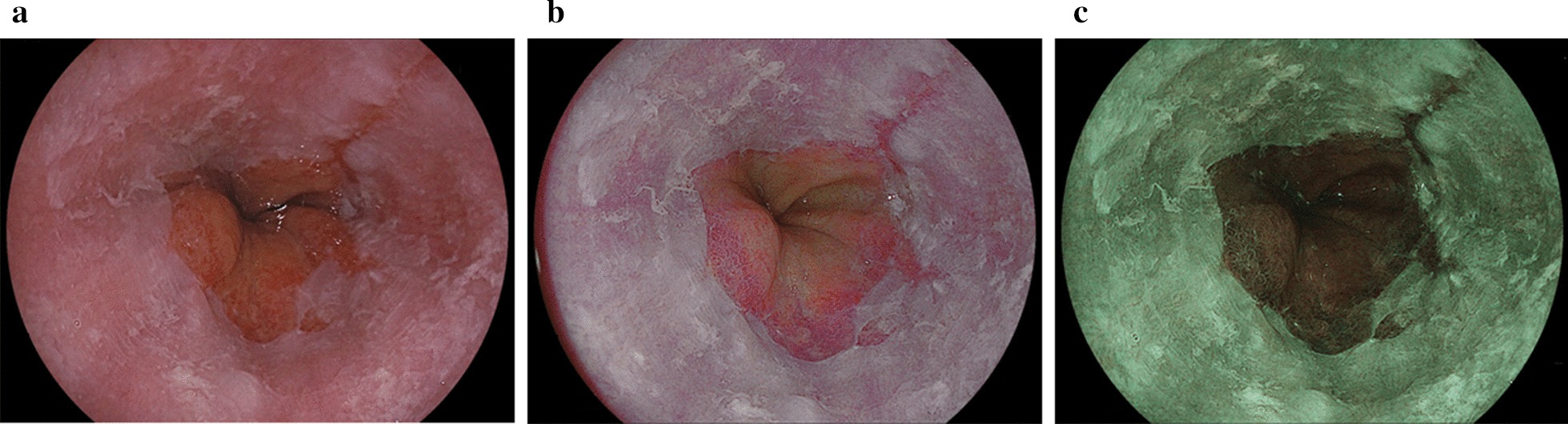
Reflux esophagitis (grade B) using white light imaging, linked color imaging, and blue LASER imaging. **a** White light imaging (WLI). Reflux esophagitis (LA grade B). **b** Linked color imaging (LCI). The reflux esophagitis was clearly highlighted in a purple–red color with LCI. The LCI image was scored as + 12 points representing improved visibility as evaluated by all endoscopists. **c** Blue LASER imaging (BLI)

**Fig. 4:**
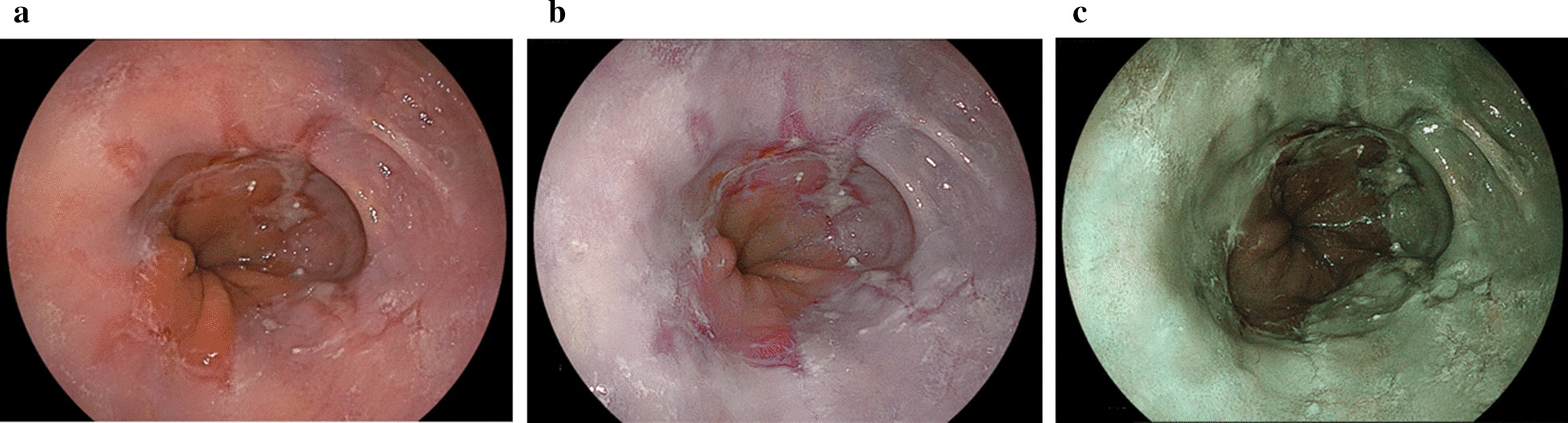
Reflux esophagitis (grade C) using white light imaging, linked color imaging, and blue LASER imaging. **a** White light imaging (WLI). Reflux esophagitis (LA grade C). **b** Linked color imaging (LCI). The reflux esophagitis (LA–C) was highlighted in a purple–red color with LCI. The LCI image was scored as + 13 points representing improved visibility as evaluated by all endoscopists. **c** Blue LASER imaging (BLI)

Images show four representative cases. Figure 1 shows endoscopic images after using WLI, LCI, and BLI. After augmenting its whitish turbidity appearance with LCI, MCE could be distinguished. Purple esophageal palisade vessels were noted with LCI when compared to WLI. All endoscopists considered the LCI image to show improved visibility, rating it as + 10 points, in agreement with trainees who assessed it as + 5 points, and experts who assessed it as + 5 points. In Fig. 2, RE (LA grade A) was detected by LCI and emphasized in a red color. The surrounding esophageal mucosa was observed with a whitish turbidity, which was emphasized with LCI. The LCI image was considered to show improved visibility and was rated at + 15 points by endoscopists, and + 6 points and + 9 points by trainees and experts, respectively. Figure 3 highlights a case of RE (LA grade B). The RE was clearly emphasized in a purple–red color with LCI. The LCI image was rated as having improved visibility and given + 12 points by all endoscopists, + 5 points by trainees, and + 7 points by experts. In Fig. 4, RE (LA grade C) was emphasized in a purple–red color with LCI. The LCI image was rated as showing improved visibility and given + 13 points by all endoscopists, + 6 points by trainees, and + 7 points by experts.

### Inter-rater reliability

ICCs with regard to inter-rater reliability and LCI versus WLI were: for MCE, 0.41 for trainees, 0.31 for experts, and 0.57 for all endoscopists; for ERE, 0.50 for trainees, 0.66 for experts, and 0.73 for all endoscopists; and for all RE; 0.47 for trainees, 0.59 for experts, and 0.71 for all endoscopists. BLI versus WLI for all endoscopists was 0.46 for MCE, 0.49 for ERE, and 0.47 for all RE (Table 4). The inter-rater reliability of LCI was: for MCE, “moderate” for trainees, “fair” for experts and “moderate” for all endoscopists; for ERE, “moderate” for trainees, “substantial” for experts, and “substantial” for all endoscopists; and for all RE, “moderate” for trainees, “moderate” for experts, and “substantial” for all endoscopists, respectively. The inter-rater reliability of BLI for MCE was “fair” for trainees and “fair” for experts; for ERE it was “slight” for trainees and “fair” for experts; and for all RE, it was “slight” for trainees and “fair” for experts, respectively.

**Table 4:** Evaluation of LCI and BLI for inter-rater reliability

Grade of reflux esophagitis								
MCE			ERE			All RE		
Trainees	Experts	All	Trainees	Experts	All	Trainees	Experts	All
ICC								
*LCI*								
0.41 (0.12–0.63)	0.31 (-0.03–0.57)	0.57 (0.37–0.72)	0.50 (0.32–0.65)	0.66 (0.54–0.76)	0.73 (0.64–0.81)	0.47 (0.31–0.59)	0.59 (0.47–0.69)	0.71 (0.63–0.78)
*BLI*								
0.34 (0.01–0.58)	0.35 (-0.05–0.56)	0.46 (0.22–0.65)	0.13 (-0.19–0.38)	0.39 (0.17–0.57)	0.49 (0.33–0.64)	0.17 (-0.68–0.39)	0.34 (0.15–0.49)	0.47 (0.33–0.59)

*BLI* blue LASER imaging, *ERE* erosive reflux esophagitis (LA grades A–D), *ICC* Intra-class correlation coefficient (95% confidence interval), *LCI* linked color imaging, *MCE* minimal change esophagitis (LA grade M)

### Intra-rater reliability

ICCs for the intra-rater reliability of LCI compared with WLI ranged from 0.43 to 0.65 for trainees and 0.43–0.69 for experts. ICCs for BLI compared with WLI ranged from 0.23 to 0.65 for trainees and 0.21–0.53 for experts (Table 5). The intra-rater reliability for LCI was “moderate–substantial” for trainees and experts. The intra-rater reliability for BLI was “slight–substantial” for trainees and “slight–moderate” for experts, respectively.

**Table 5:** Evaluation of LCI and BLI for intra-rater reliability

Image	Trainees (n: 5)					Experts (n: 5)				
	A	B	C	D	E	A	B	C	D	E
*ICC*										
LCI	0.46 (0.24–0.61)	0.65 (0.51–0.75)	0.43 (0.21–0.59)	0.56 (0.38–0.68)	0.43 (0.21–0.59)	0.43 (0.20–0.59)	0.45 (0.23–0.60)	0.67 (0.55–0.78)	0.59 (0.44–0.71)	0.69 (0.57–0.78)
BLI	0.65 (0.51–0.75)	0.23 (-0.07–0.45)	0.43 (0.21–0.59)	0.43 (0.21–0.59)	0.24 (-0.06–0.45)	0.21 (-0.11–0.43)	0.53 (0.35–0.67)	0.21 (-0.10–0.43)	0.31 (0.03–0.50)	0.34 (0.09–0.52)

*BLI* blue LASER imaging, *ICC* intra-class correlation coefficient (95% confidence interval), *LCI* linked color imaging

### Objective evaluations

**Fig. 5:**
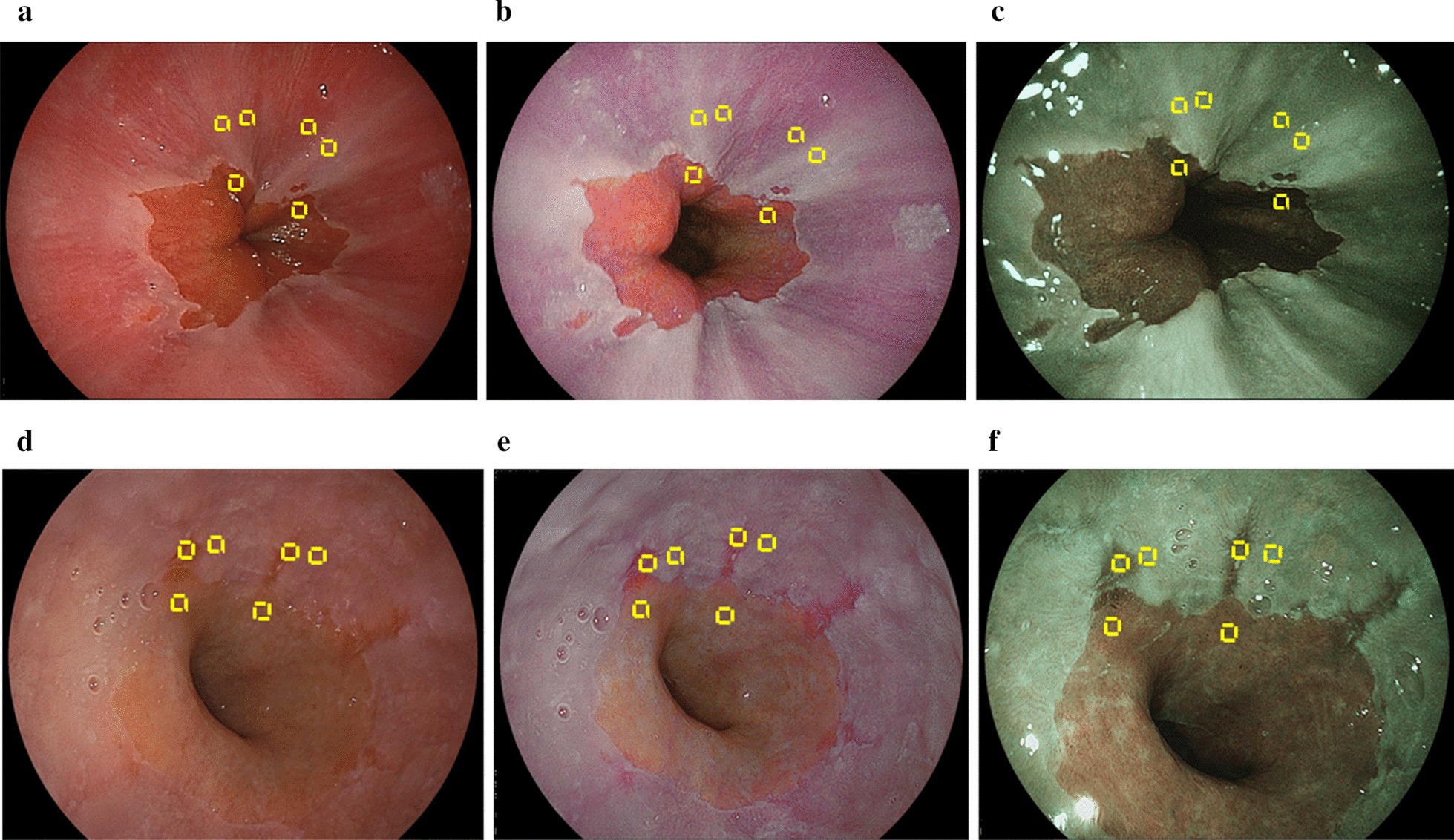
Representative endoscopic images captured using **a** white light (WLI) or **b** linked color imaging (LCI) or **c** blue LASER imaging (BLI) in minimal change esophagitis (MCE); and **d** WLI or **e** LCI or **f** BLI in reflux esophagitis (LA grade A). Broken yellow lines delineate regions of interest (ROIs; 20 × 20 pixels). ROIs in adjacent esophageal, reflux esophagitis, and gastric mucosae were fixed in identical positions for each specific lesion in all images

Representative endoscopic images using WLI, LCI, and BLI with ROIs are shown in Fig. 5. Calculations for L* a* b* color values for adjacent esophageal, RE, and gastric mucosae were made in MCE and ERE (Table 6). WLI and LCI showed significant differences, but not for L* in the esophageal mucosa, a* in the gastric mucosa for MCE, and a* in the RE mucosa for ERE.

**Table 6:** Objective evaluations using L*, a*, and b* color values (mean ± SD)

Reflux esophagitis	L*, a* b* values	WLI	LCI	BLI	*P* value	
					WLI vs. LCI	WLI vs. BLI
*Surrounding Esophageal mucosa*						
MCE	L*	40.3 ± 6.9	39.6 ± 9.1	50.8 ± 7.9	n.s	< 0.001
	a*	27.8 ± 4.1	23.5 ± 5.2	– 15.8 ± 3.2	< 0.001	< 0.001
	b*	18.4 ± 3.2	– 0.7 ± 4.4	14.3 ± 1.4	< 0.001	< 0.001
ERE	L*	45.5 ± 8.1	56.9 ± 11.9	49.5 ± 10.5	< 0.001	< 0.01
	a*	21.6 ± 4.6	10.0 ± 6.0	– 14.5 ± 4.3	< 0.001	< 0.001
	b*	13.4 ± 4.6	– 0.8 ± 3.0	13.7 ± 2.3	< 0.001	n.s
*RE mucosa*						
MCE	L*	44.7 ± 5.7	52.9 ± 9.4	60.9 ± 12.2	< 0.001	< 0.001
	a*	20.0 ± 3.3	8.3 ± 3.9	– 17.5 ± 2.4	< 0.001	< 0.001
	b*	13.5 ± 3.5	– 0.2 ± 2.3	12.8 ± 2.4	< 0.001	n.s
ERE	L*	35.4 ± 6.6	40.0 ± 9.6	28.1 ± 8.3	< 0.001	< 0.001
	a*	28.8 ± 5.3	28.2 ± 9.6	– 5.9 ± 3.7	n.s	< 0.001
	b*	24.6 ± 6.1	6.9 ± 7.9	12.1 ± 2.4	< 0.001	< 0.001
*Gastric mucosa*						
MCE	L*	32.6 ± 6.6	42.3 ± 5.6	30.1 ± 11.6	< 0.001	n.s
	a*	23.6 ± 4.2	22.1 ± 4.4	– 2.0 ± 3.0	n.s	< 0.001
	b*	22.9 ± 5.1	15.6 ± 4.5	12.9 ± 3.7	< 0.001	< 0.001
ERE	L*	32.5 ± 8.4	42.2 ± 6.1	19.3 ± 8.9	< 0.001	< 0.001
	a*	24.8 ± 6.0	21.3 ± 4.6	– 0.3 ± 3.5	< 0.001	< 0.001
	b*	23.3 ± 6.7	16.6 ± 9.4	10.9 ± 3.8	< 0.001	< 0.001

*BLI* blue LASER imaging, *ERE* erosive reflux esophagitis (LA grades A–D), *LCI* linked color imaging, *MCE* minimal change esophagitis (LA grade M), *n.s.* not significant, *RE* reflux esophagitis, *SD* standard deviation, *WLI* white light imaging

Table 7 outlines objective evaluations based on Δ L*, Δ a*, and Δ b*, and color differences (ΔE*). The ΔE* values were as follows: WLI: 12.3, LCI: 22.7, BLI: 13.5 in MCE; and WLI: 18.2, LCI: 31.9, BLI: 24.1 in ERE for surrounding esophageal and RE mucosae**;** WLI: 17.6, LCI: 25.8, BLI: 35.2 in MCE; and WLI: 11.5, LCI: 20.3, BLI: 12.8 in ERE for RE and gastric mucosae. For both the adjacent surrounding esophageal mucosa vs. RE mucosa and RE mucosa vs. gastric mucosa, statistically significant differences in the color difference (ΔE*) between WLI and LCI (*P* < 0.001) for both MCE and ERE were noted. A significant difference was not observed between LCI and BLI used in MCE for the surrounding esophageal mucosa vs. RE mucosa, and in ERE for the RE mucosa vs. gastric mucosa.

**Table 7:** Objective evaluations using color differences (Δ L*, Δ a*, Δ b*, Δ E*; mean ± SD)

Reflux esophagitis	L*, a* b* values	WLI	LCI	BLI	*P* value		
					WLI vs. LCI	WLI vs. BLI	LCI vs. BLI
*Surrounding esophageal mucosa vs. RE mucosa*							
MCE	∆L*	4.4 ± 6.5	13.3 ± 9.5	10.2 ± 10.6	< 0.001	< 0.01	n.s
	∆a*	– 7.8 ± 3.2	– 15.2 ± 6.9	– 1.7 ± 2.7	< 0.001	< 0.001	< 0.001
	∆b*	– 4.9 ± 3.2	0.5 ± 4.5	– 1.5 ± 2.6	< 0.001	< 0.001	< 0.01
	∆E*	12.3 ± 3.9	22.7 ± 7.1	13.5 ± 7.3	< 0.001	n.s	< 0.001
ERE	∆L*	– 10.8 ± 5.9	– 16.9 ± 12.9	– 21.5 ± 9.1	< 0.001	< 0.001	< 0.01
	∆a*	7.3 ± 4.9	18.7 ± 13.9	8.6 ± 4.4	< 0.001	n.s	< 0.001
	∆b*	11.2 ± 6.3	7.8 ± 7.7	– 1.6 ± 3.3	< 0.01	< 0.001	< 0.001
	∆E*	18.2 ± 6.8	31.9 ± 8.5	24.1 ± 8.4	< 0.001	< 0.001	< 0.001
*RE mucosa vs. Gastric mucosa*							
MCE	∆L*	– 12.1 ± 6.9	– 10.6 ± 10.0	– 30.8 ± 11.2	n.s	< 0.001	< 0.001
	∆a*	3.6 ± 3.6	13.8 ± 5.0	15.4 ± 3.4	< 0.001	< 0.001	n.s
	∆b*	9.4 ± 5.3	15.8 ± 4.7	0.2 ± 4.9	< 0.001	< 0.001	< 0.001
	∆E*	17.6 ± 5.4	25.8 ± 5.9	35.2 ± 10.6	< 0.001	< 0.001	< 0.001
ERE	∆L*	– 2.9 ± 8.6	2.2 ± 10.1	– 8.7 ± 8.9	< 0.001	< 0.001	< 0.001
	∆a*	– 4.0 ± 5.6	– 6.9 ± 10.8	5.6 ± 3.9	< 0.05	< 0.001	< 0.001
	∆b*	– 1.2 ± 6.5	9.7 ± 12.6	– 1.2 ± 2.9	< 0.001	n.s	< 0.001
	∆E*	11.5 ± 6.4	20.3 ± 10.7	12.8 ± 6.9	< 0.001	n.s	< 0.001

*BLI* blue LASER imaging, *ERE* erosive reflux esophagitis (LA grades A–D), *LCI* linked color imaging, *MCE* minimal change esophagitis (LA grade M), *RE* reflux esophagitis, *SD* standard deviation, *WLI* white light imaging, ΔE* color difference (ΔE* = [(ΔL*) ^2^ + (Δa*) ^2^ + (Δb*) ^2^]^1/2^)

## Discussion

WLI allows the easy diagnosis of severe ERE in contrast to the more difficult identification of MCE or a mild case of ERE. In Japan, the frequency of severe RE has been low but that of mild cases is high [34]. In recent years, Deng et al. reported the improved detection of MCE in non-erosive reflux esophagitis [35]. However, whether the visibility of endoscopic findings of RE is improved by using LCI compared to WLI continues to be unclear. We are the first to evaluate LCI and BLI in RE using subjective and objective analyses.

We herein sought to assess the visibility and inter- and intra-rater reliability of LCI and BLI, and compare this with WLI, in detecting RE using visibility scores and ICCs. All endoscopists yielded total visibility scores of 35.7 for MCE, 37.6 for ERE, and 36.9 for all RE. Trainees and experts did not show significantly different visibility scores (Table 2). We thus conclude that LCI might improve diagnoses made by both experts and trainees. For ERE, purple-red or more reddish colors were emphasized in LCI while surrounding esophageal mucosa (whitish turbidity) was observed to be more whitish leading us to conclude that increased visibility was achieved. In a sub-analysis of LCI users and non-users, visibility scores were significantly higher for LCI users (*P* < 0.001). We postulate that visibility will improve with increasing experience of LCI, regardless if users are experts or trainees.

By contrast, improved visibility of RE using BLI was not observed even though narrow band imaging (NBI) has been reported to improve the visibility of RE [11]. However, since most of these studies used magnifying endoscopy or close-up observations, further detailed observations may result in an improvement of visibility with BLI in future. BLI did not appear to improve visibility for trainees and experts in our study because the BLI color tone of RE looked similar to that of the background mucosal surface.

A comparison of the inter-rater reliability of LCI with WLI yielded the following ICCs: 0.57 for MCE, 0.73 for ERE, and 0.71 for all RE. LCI showed an inter-rater reliability that was “substantial” for ERE and “moderate” for MCE, while the intra-rater reliability for LCI was “moderate–substantial” for trainees and experts. MCE showed reduced ICC values compared to those of ERE. Low improved visibility with MCE compared to ERE may be caused by low inter-rater reliability with MCE. The inter-observer agreement for a diagnosis of MCE was found to be poor using WLI by others [36]. Deng et al. [35] reported that LCI improved the detection of MCE in non-erosive RE, which indicates that LCI may lead to an improvement in inter-rater reliability for the diagnosis of MCE.

ICC values for non-magnified BLI were lower than those for LCI. Lee et al. [37] reported that the inter-observer agreement for grading RE was substantial when non-magnified NBI was used with WLI. According to previous studies [11, 38], the inter-observer agreement for magnified NBI in RE was “very good” for visibility of mucosal morphology, which indicates that combined or magnified observations with BLI may lead to an improved inter-observer agreement with BLI.

L* a* b* color values, as well as those of the ΔE*, in adjacent esophageal and RE mucosae were calculated objectively. L* was found to be significantly higher for LCI when compared to WLI for RE mucosa**.** Increasing L* values for LCI indicate this was lighter than WLI, which is one of the causes of improved visibility compared to WLI. The Δa* was higher for LCI compared to WLI in ERE between the surrounding and RE mucosae, which suggests that the RE mucosa became highlighted in a red color with LCI. Additionally, the color difference was significantly different when comparing WLI and LCI for both MCE and ERE. These results indicate that using LCI leads to a clearer observation of RE and improved contrasting images for both ERE and MCE. In contrast, ΔE* was not significantly different when comparing WLI and BLI in MCE between surrounding and RE mucosae, and for ERE between RE and gastric mucosae, which is one of the causes for no improved visibility in BLI compared to WLI.

However, we acknowledge several limitations within our study. First, the number of patients used was small, especially those with RE of LA grades B, C, and D, for which a sample size of 52 was not reached, and were from a single center. Second, since visibility was evaluated subjectively, this may have introduced observer bias, leading us to undertake objective quantitative analyses to ensure absolute evaluations of each image. Third, we did not attempt to elucidate any association between visibility and histological diagnosis. Thus, our findings need to be confirmed in a prospective study using a larger number of patients and histological examinations.

## Conclusion

In conclusion, LCI increases the visibility of RE by enhancing contrast for images of both MCE and ERE when compared to WLI and BLI. The ICC for LCI was “substantial” for all RE, and the visibility and ICC for MCE were lower than for ERE using LCI. Our results indicate that LCI can lead to an improved diagnosis of MCE and ERE.

## Data Availability

The datasets and materials used during the current study are available from the corresponding author on reasonable request.

## Abbreviations

RE: Reflux esophagitis
LCI: Linked color imaging
WLI: White light imaging
BLI: Blue LASER imaging
ICC: Intra-class correlation coefficient
GERD: Gastroesophageal reflux disease
IEE: Image-enhanced endoscopy
EGD: Esophagogastroduodenoscopy
GE-J: Gastroesophageal junction
SC-J: Squamocolumnar junction
MCE: Minimal change esophagitis
ERE: Erosive reflux esophagitis
ROI: Region of interest
CIs: Confidence intervals
